# Sofosbuvir induces gene expression for promoting cell proliferation and migration of hepatocellular carcinoma cells

**DOI:** 10.18632/aging.204170

**Published:** 2022-07-12

**Authors:** Wei-Lun Tsai, Jin-Shiung Cheng, Pei-Feng Liu, Tsung-Hsien Chang, Wei-Chih Sun, Wen-Chi Chen, Chih-Wen Shu

**Affiliations:** 1 Division of General Internal Medicine, Department of Internal Medicine, Kaohsiung Veterans General Hospital, Kaohsiung, Taiwan; 2 Division of Gastroenterology and Hepatology, Department of Internal Medicine, Kaohsiung Veterans General Hospital, Kaohsiung, Taiwan; 3 School of Medicine, National Yang Ming Chiao Tung University, Taipei, Taiwan; 4 School of Nursing, Fooyin University, Kaohsiung, Taiwan; 5 Department of Biomedical Science and Environmental Biology, Kaohsiung Medical University, Kaohsiung, Taiwan; 6 Department of Medical Research, Kaohsiung Medical University Hospital, Kaohsiung, Taiwan; 7 Department and Graduate Institute of Microbiology and Immunology, National Defense Medical Center, Taipei, Taiwan; 8 Institute of BioPharmaceutical Sciences, National Sun Yat-sen University, Kaohsiung, Taiwan

**Keywords:** hepatitis C, hepatocellular carcinoma, sofosbuvir

## Abstract

Direct-acting antivirals (DAAs) have achieved a sustained virological response (SVR) rate of 95–99% in treating HCV. Several studies suggested that treatment with sofosbuvir (SOF), one type of DAAs, may be associated with increased risk of developing HCC. The aim of this study is to investigate the potential mechanisms of SOF on the development of HCC. OR-6 (harboring full-length genotype 1b HCV) and Huh 7.5.1 cells were used to examine the effects of SOF on cell proliferation and migration of HCC cells. SOF-upregulated genes in OR-6 cells were inspected using next generation sequencing (NGS)and the clinical significance of these candidate genes was analyzed using The Cancer Genome Atlas (TCGA) database. We found that SOF increased cell proliferation and cell migration in OR-6 and Huh 7.5.1 cells. Several SOF-upregulated genes screened from NGS were confirmed by real-time PCR in OR-6 cells. Among these genes, PHOSPHO2, KLHL23, TRIM39, TSNAX-DISC1 and RPP21 expression were significantly elevated in the tumor tissues compared with the non-tumor tissues of HCC according to TCGA database. High expression of PHOSPHO2 and RPP21 was associated with poor overall survival of HCC patients. Moreover, knockdown of PHOSPHO2-KLHL23, TSNAX-DISC1, TRIM39 and RPP21 diminished cell proliferation and migration increased by SOF in OR-6 and Huh 7.5.1 cells. In conclusion, SOF-upregulated genes promoted HCC cell proliferation and migration, which might be associated with the development of HCC.

## INTRODUCTION

The prevalence rate of hepatitis C virus (HCV) infection is around 2–5% in Taiwan. HCV is a major etiology of cirrhosis and hepatocellular carcinoma (HCC) in the world [1]. In patients who acquired acute HCV infection, 60–90% will become chronic HCV infection and cirrhosis and HCC will develop in 20–30% of patients after 20–30 years of infection [2, 3]. In recent years, anti-HCV therapy has made a tremendous progress. The development of *in vitro* cell culture systems and the knowledge of the life cycle of HCV, has led to the discovery of a number of targets for direct-acting antiviral (DAA) agents [4, 5]. Several DAAs targeting different families of the virus including NS5B nucleotide inhibitors (NI), NS5A complex inhibitors and NS3/4A protease inhibitors (PI)] have been developed [6–9]. Sofosbuvir (SOF)-based regimen has been most widely used for the treatment of chronic hepatitis C (CHC) and has a sustained virological response (SVR) rate of 95–99% in compensated cirrhosis and 85–95% in patients with decompensated liver cirrhosis in recent studies [2, 10–17].

However, some evidence showed that the DAA treatment did not eradicate the development of HCC in these CHC patients [18–20]. A recent study by Reig et al., discovered that in 103 CHC patients with history of HCC who received DAA treatment, 16 (27.6%) of patients developed GCC recurrence after a median follow-up time of 5.7 months [21]. In another recent study, Conti et al., discovered that among 344 cirrhotic patients who received DAA, HCC was found in 26 (7.6%) patients. Among them, 17 of 59 patients (28.81%) had history of HCC and 9 of 285 patients (3.16%) without history of HCC developed HCC [22]. From a Spanish study in the 70 CHC patients with history of HCC, recurrence of HCC happened in 21 (30%) within 12 months of starting DAA treatment [23]. However, there are also some studies that did not show an increased recurrence of HCC following DAAs therapy [24–26]. Nevertheless, most of these studies are retrospectively designed and no randomized controlled studies have ever been performed up to now owing to ethical concern. The issue remains controversial.

A recent study has found that DAA treatment causes an early increase in serum VEGF level and the inflammatory pattern may change, during DAA treatment, but such changes may reverse to normal after treatment [27]. Another recent study also found that SOF activates an increase in EGFR expression and phosphorylation in hepatoma cells [28]. Another recent study has revealed association between cytokine levels and the development of HCC in CHC patients who received DAAs [29]. In a study by Chu et al., on-treatment decrease of NKG2D was found to be a useful predictor of early development of HCC in CHC patients who received DAA treatment [30]. In another study, Hengst et al., also found that chronic HCV infection may interfere with the milieu of soluble inflammatory mediators after HCV clearance and HCV cure did not lead to complete immunological change [31].

Collectively, current studies have discovered that DAA treatment for HCV may result in changes in inflammatory microenvironment that was related to happening of HCCs that can partly explain the increased development of HCC in some studies. However, studies that tried to discover the host factors related to the development of HCC after DAA treatment are little. In a recent study, Perez et al., discovered that HCV may cause epigenetic changes that reprogram host gene expression and these findings persist following HCV elimination [32]. These may explain why some CHC patients could develop HCC after HCV elimination. However, there has been no studies aimed to investigate the impact of SOF-upregulated genes on the happening of HCC in CHC patients. In this study we found that SOF increased cell proliferation and migration in HCC cells. Several SOF-upregulated genes discovered by next-generation sequencing (NGS) were related with the development of HCC according to The Cancer Genome Atlas (TCGA) database; and among them, several genes were related with overall and disease-free survival. Knockdown of these genes reduced cell proliferation and migration increased by SOF in HCC cells, implying that SOF might have risks in the occurrence of HCC through upregulation of certain genes.

## RESULTS

### Sofosbuvir (SOF) increased cell proliferation and migration in hepatoma cells

**Figure 1:**
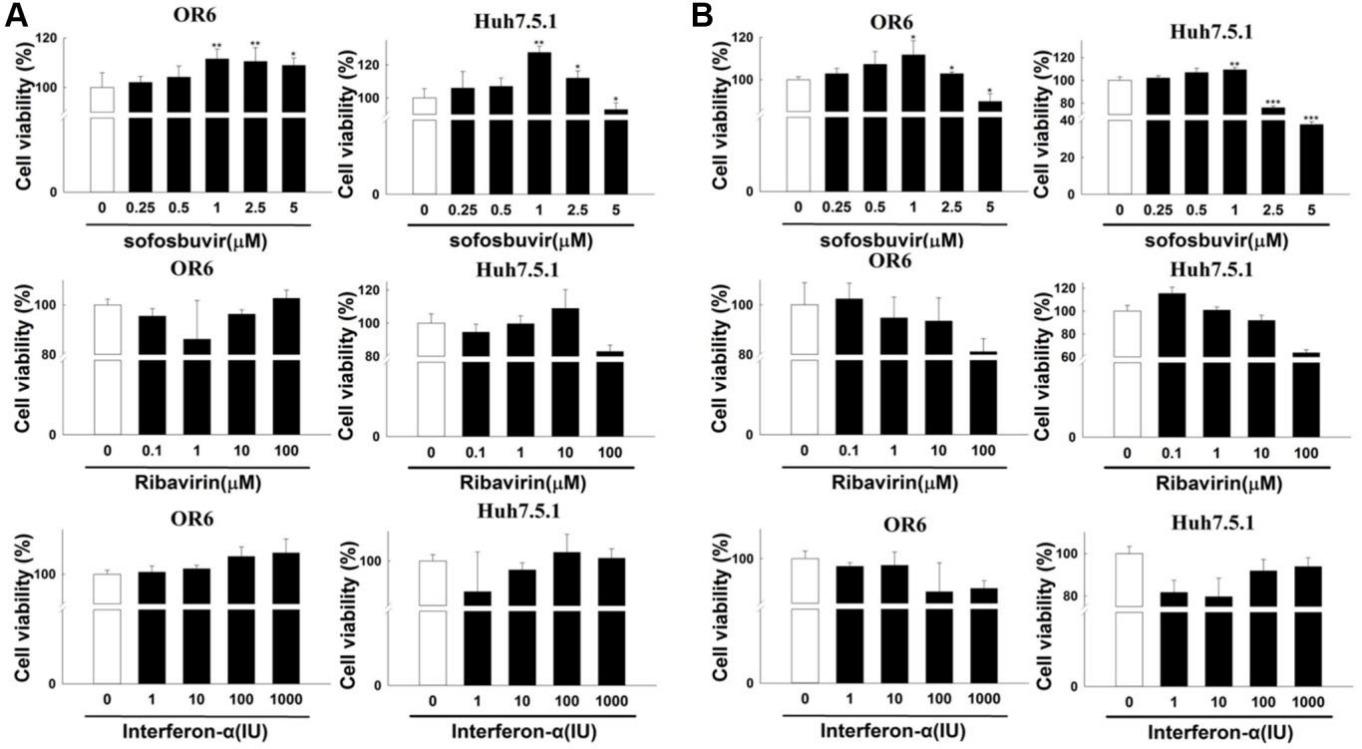
**The effects of sofosbuvir, ribavirin and interferon-α on cell proliferation of HCC cells.** (**A**) Two-dimensional cell proliferation assay in OR-6 and Huh 7.5.1 cells treated with different doses of SOF (0.25 to 5 μM), interferon-α (1 to 1000 IU) and ribavirin (0.1 to 100 μM) for 24 hrs were performed. (**B**) The OR-6 and Huh 7.5.1 cells were grown in three-dimensional culture dish and treated with different doses of SOF (0.25 to 5 μM), interferon-α (1 to 1000 IU) and ribavirin (0.1 to 100 μM) for 24 hrs to inspect cell proliferation. The experiments were performed from three independent experiments. (^*^p < 0.05; ^**^p < 0.01; ^***^p < 0.001).

SOF-based regimen has been found to be related with the occurrence or recurrence of HCC especially in CHC patients with history of HCC [21–23]. Enhanced cell proliferation is a common feature for the development of HCC. Huh 7.5.1 cells and OR-6 cells were used in the experiments. The influence of SOF on cell proliferation was examined in this study. In OR-6 and Huh 7.5.1 cells treated with different doses of SOF (0.25 to 5 μM) for 24 h, SOF increased cell proliferation in OR-6 and Huh 7.5.1 cells in two- and three-dimensional culture models (Figure 1A and 1B, respectively). Interferon-αand ribavirin combination therapy are regarded as the standard of care for CHC patients before the development of DAA. However, in CHC patients who received interferon-α with or without ribavirin treatment did not show an increased risk of the development of HCC in many previous studies [33–36]. Interferon-α and ribavirin served as controls in this study. In OR-6 and Huh 7.5.1 cells treated with different doses of interferon-α (1 to 1000 IU) and ribavirin (0.1 to 100 μM) for 24 h, two- and three-dimensional cell proliferation were not significantly influenced (Figure 1A and 1B, respectively).

**Figure 2:**
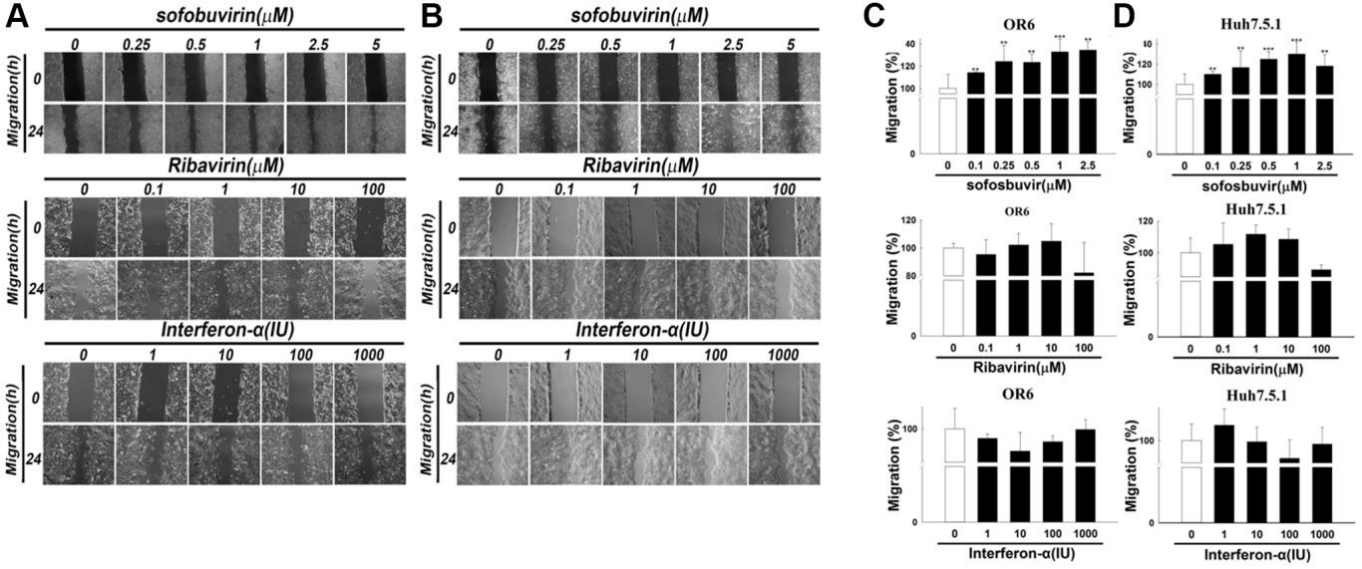
**Cell migration assay in sofosbuvir treated hepatoma cells using Interferon-α and ribavirin as controls.** Cell migration assay was performed in OR-6 cells (**A**) and Huh 7.5.1 cells (**B**) treated with different doses of SOF (0.1 to 2.5 μM), interferon-α (1 to 1000 IU) and ribavirin (0.1 to 100 μM) for 24 hrs in the left panel. The quantitative results for migratory distance of (**C**) OR-6 cells or (**D**) Huh 7.5.1 were calculated with ImageJ software and expressed as the mean ± SEM from three independent experiments. (^*^p < 0.05; ^**^p < 0.01; ^***^p < 0.001).

Moreover, cancer cell migration is the typical feature involved in cancer metastasis. The effects of SOF treatment on cell migration were investigated in this study. In OR-6 and Huh 7.5.1 cells treated with different doses of SOF (0.10 to 2.5 μM) for 24 h, cell migration was increased (Figure 2A–2D, respectively) in HCC cells exposed to SOF. Interferon-α and ribavirin served as controls in this study. In OR-6 and Huh 7.5.1 cells treated with different doses of interferon-α (1 to 1000 IU) and ribavirin (0.1 to 100 μM) for 24 h, cell migration was not influenced (Figure 2A and 2B, respectively). We have also performed the experiments in AML-12 cells (non-HCC cancer-derived cell lines) and were shown in Supplementary Figure 1A–1C. We discovered that SOF increased cell proliferation and migration in AML-12 cells as well as in OR-6 cells. We have performed cell viability and migration assay of daclatasvir and entecavir in OR-6 cells and we found that daclatasvir but not entecavir slightly increased cell proliferation but daclatasvir and entecavir had no influences on migration assay in OR-6 cells (Supplementary Figure 2A and 2B).

### SOF-upregulated genes were related with cancer development of HCC

Next-generation sequencing (NGS) of the complete RNA transcriptome (RNA-seq) is a novel method to characterize the molecular mechanism of diseases [37–40]. In OR-6 cells treated with SOF 1 μM for 24 hours, NGS study was performed and many genes were upregulated by SOF in OR-6 cells. Top-ranked genes were selected for further investigation (Supplementary Table 1). To further evaluate the clinical meaning of the upregulated genes in patients with HCC, the TCGA database, one of the largest genomic databases available for various cancer types that can be downloaded from an open-access resource [41–43], is used for analysis (Table 1).The TCGA data analysis showed that several genes upregulated by SOF in OR-6 cells, including PHOSPHO2, KLHL23, TSNAX-DISC1, TRIM39 and RPP21, which were significantly higher in HCC tumor than in non-tumor parts (Table 1), suggesting that PHOSPHO2, KLHL23, TSNAX-DISC1, TRIM39 and RPP21 were associated with the development of HCC. The association of survival with these SOF-upregulated genes were analyzed by univariate and multivariate Cox proportional hazards model. Statistical analysis of the TCGA database found that PHOSPHO2 (Adjusted hazard ratio (AHR) was 1.72, 95% confidence interval (CI) was 1.08–2.51 and *p*-value was 0.005) and RPP21 (AHR was 1.49, 95% CI was 1.02–2.18, and *p*-value was 0.040) were associated with overall survival of HCC, whilePHOSPHO2 (AHR was 1.59, 95% CI was 1.07–2.36 and *p*-value was 0.021) was associated with disease-free survival in patients with HCC (Table 2).

**Table 1:** Comparison of SOF up-regulated genes in tumor versus non-tumor parts in HCC.

**Variables**	**Non-tumor part**	**Tumor part**	*p*-value^*^
	**Mean ± SD**	**Mean ± SD**	
PHOSPHO2	4,84 ± 0.67	5.44 ± 1.07	<0.001
KLHL23	5.67 ± 1.03	6.25 ± 2.23	0.049
TSNAX-DISC1	0.10 ± 0.34	0.68 ± 1.79	0.017
TRIM39	8.12 ± 0.21	8.51 ± 0.51	<0.001
RPP21	8.14 ± 0.45	8.61 ± 0.76	<0.001

**Table 2:** Associated of SOF up-regulated genes with overall survival and disease-free survival of HCC patients.

**Variable**		**No. (%)**	**CHR (95% CI)**	*p* value^*^	**AHR (95% CI)**	***p* value^†^**
**Overall survival**						
PHOSPHO2	Low	242 (68.2)	1.00		1.00	
	High	113 (31.8)	**1.75 (1.23–2.49)**	**0.002**	**1.72 (1.18–2.51)**	**0.005**
KLHL23	Low	133 (37.5)	1.00		1.00	
	High	222 (62.5)	**1.50 (1.03–2.19)**	**0.034**	1.37 (0.93–2.03)	0.115
TSNAX-DISC1	Low	286 (80.6)	1.00		1.00	
	High	69 (19.4)	1.08 (0.70–1.68)	0.720	0.97 (0.60–1.58)	0.907
TRIM39	Low	247 (69.6)	1.00		1.00	
	High	108 (30.4)	1.31 (0.91–1.89)	0.148	1.32 (0.90–1.95)	0.157
RPP21	Low	248 (69.9)	1.00		1.00	
	High	107 (30.1)	1.28 (0.89–1.83)	0.190	**1.49 (1.02–2.18)**	**0.040**
**Disease-free survival**						
PHOSPHO2	Low	230 (74.2)	1.00		1.00	
	High	80 (25.8)	1.43 (0.99–2.06)	0.059	**1.59 (1.07–2.36)**	**0.021**
KLHL23	Low	113 (36.5)	1.00		1.00	
	High	197 (63.5)	1.43 (0.99–2.05)	0.054	1.44 (0.99–2.11)	0.060
	High	8 (2.6)	1.37 (0.56–3.35)	0.491	1.01 (0.37–2.77)	0.979
TSNAX-DISC1	Low	249 (80.3)	1.00		1.00	
	High	61 (19.7)	1.23 (0.82–1.85)	0.311	1.27 (0.83–1.94)	0.277
	High	59 (19.0)	1.24 (0.83–1.85)	0.294	1.23 (0.81–1.87)	0.338
TRIM39	Low	272 (87.7)	1.00		1.00	
	High	38 (12.3)	1.20 (0.75–1.91)	0.441	0.97 (0.59–1.62)	0.914
RPP21	Low	165 (53.2)	1.00		1.00	
	High	145 (46.8)	1.37 (0.97–1.92)	0.071	1.31 (0.92–1.86)	0.142

Abbreviations: HCC: hepatocellular carcinoma; CHR: crude hazard ratio; CI: confidence interval; AHR: adjusted hazard ratio; AJCC: American Joint Committee on Cancer; ^†^*p* values were adjusted for cell differentiation (moderate + poor vs. well) and AJCC pathological stage (stage III + IV vs. stage I + II) by multivariate Cox’s regression.

### Silencing upregulated genes reduced SOF-mediated cell proliferation and migration

**Figure 3:**
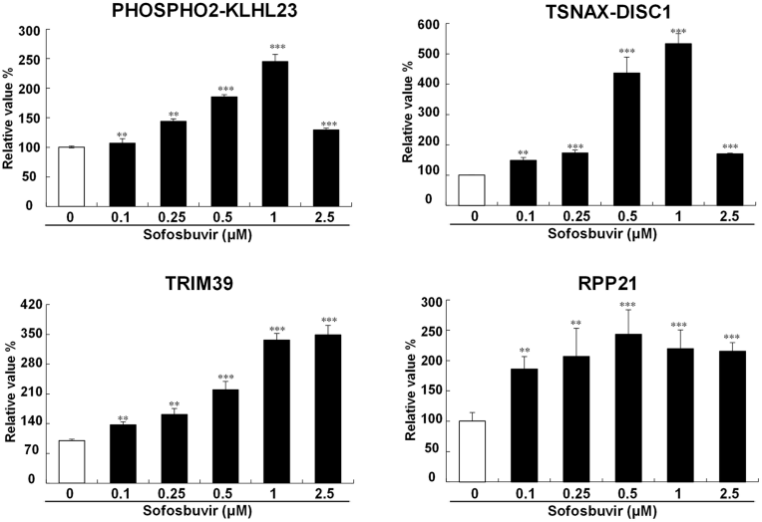
**Validation of candiate genes up-regulated by SOF.** To avalidate the SOF-upregulated genes identified from NGS, the OR-6 cells were treated with SOF (0.1 to 2.5 μM) for 24 hrs. The cells were harvested to determine gene expression with Q-PCR validation of PHOSPHO2-KLHL23, TSNAX-DISC1, TRIM39, RPP21 in OR-6 cells were performed using GAPDH as a normalized control. The experiments were performed from three independent experiments. (^*^p < 0.05; ^**^p < 0.01; ^***^p < 0.001).

NGS studies revealed that several genes including PHOSPHO2-KLHL23, TSNAX-DISC1, TRIM39, RPP21 were upregulated by SOF in OR-6 cells and were related with the occurrence of HCC according to the TCGA database. These upregulated genes in OR-6 and Huh 7.5.1 cells were analyzed using Q-PCR. Our results discovered that PHOSPHO2-KLHL23, TSNAX-DISC1, TRIM39 and RPP21 were significantly upregulated by SOF in OR-6 cells and Huh 7.5.1 cells (Supplementary Figure 3 and Figure 3). Of note, the expression levels of phospho2-KLH23 and TSNAX-DISC1 were increased upon sofosbuvir treatment in a dose dependent manner and peaked at 1 μM of SOF. From the point of pharmacology, the effects of SOF may reach a plateau at a concentration of 1 μM and subsequently decreased at a concentration of 2.5 μM, which might be a normal pharmacological phenomenon.

**Figure 4:**
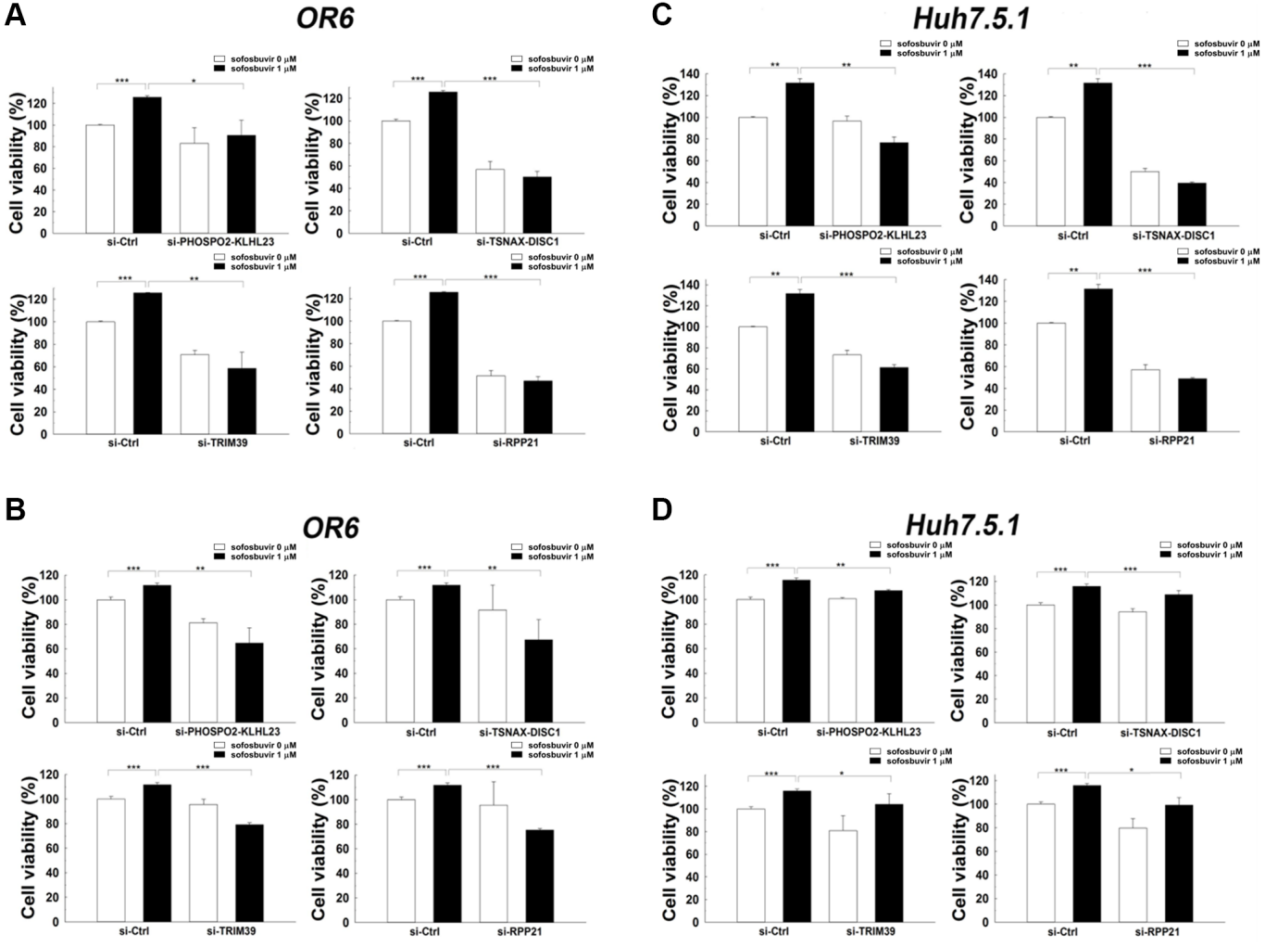
**Effects of siRNA against candidate genes on cell proliferation assay in SOF treated OR-6 and Huh 7.5.1 cells.** OR-6 (**A**, **B**) and Huh 7.5.1 (**C**, **D**) cells were transfected with non-targeting siRNA (si-Ctrl) or siRNA against PHOSPHO2-KLHL23, TSNAX-DISC1, TRIM39 and RPP21for 48 hrs. The cells were then treated with SOF 1 μM for 24 hrs. The ATP level in cells were accessed to measure cell proliferation (**A**, **C**). In addition, the cells were seeded on 3D culture plates to form tumor sphere in the presence or absence of 1 μM SOF for 24 hrs. The experiments were performed from three independent experiments. (^*^p < 0.05; ^**^p < 0.01; ^***^p < 0.001).

Previous results showed that SOF increased two- and three-dimensional cell proliferation in hepatoma cells with or without HCV infection (Figure 1A and 1B, respectively). Furthermore, PHOSPHO2-KLHL23, TSNAX-DISC1, TRIM39 and RPP21, which were upregulated by SOF in OR-6 cells, were associated with the occurrence of HCC according to the analysis of the TCGA database (Table 2). The effects of these genes on SOF-enhanced cell proliferation remained unknown. siRNA mediated knockdown of PHOSPHO2-KLHL23, TSNAX-DISC1, TRIM39 and RPP21 improved SOF-elevated cell proliferation in OR-6 and Huh 7.5.1 cells in both two- and three-dimensional cultures (Figure 4). The present results indicated that PHOSPHO2-KLHL23, TSNAX-DISC1, TRIM39 and RPP21 were associated with cell proliferation in SOF-treated hepatoma cells with and without HCV infection.

**Figure 5:**
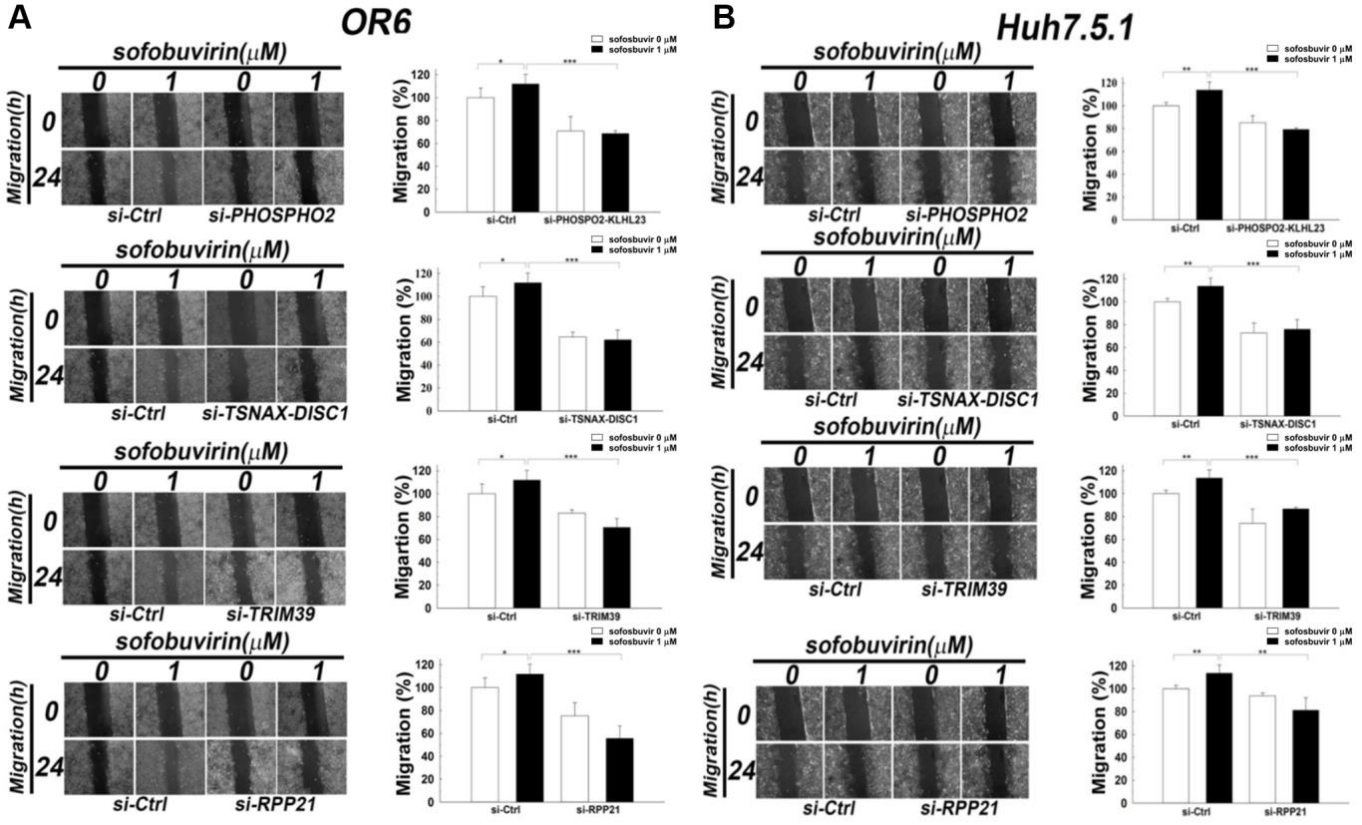
**Effects of siRNA against candidate genes on cell migration in SOF treated OR-6 and Huh 7.5.1 cells.** OR-6 (**A**) and Huh 7.5.1 cells (**B**) were transfected with non-targeting siRNA (si-Ctrl) or siRNA against PHOSPHO2-KLHL23, TSNAX-DISC1, TRIM39 and RPP21 for 48 hrs. The cells were seeded on well chamber and treated with SOF 1 μM for 24 hrs to determine the effects of the genes in migration activity. The same control (siCtrl) is shown to compare differential migratory effects of each gene silencing in cells with or without SOF treatment. The migratory distance of the cells was quantified with ImageJ software and expressed as the mean ± SD from three independent experiments. (^*^p < 0.05; ^**^p < 0.01; ^***^p < 0.001).

In addition, the association of these genes with SOF-enhanced cell migration also remained unknown. Knockdown of PHOSPHO2-KLHL23, TSNAX-DISC1, TRIM39 and RPP21using corresponding siRNA indicated that the increase in cell migration by SOF was reduced in OR-6 and Huh 7.5.1 cells when transfected with siRNA against these genes (Figure 5). Current findings suggested that PHOSPHO2-KLHL23, TSNAX-DISC1, TRIM39 and RPP21 were associated with cell migration in SOF-treated hepatoma cells with and without HCV infection.

## DISCUSSION

Direct-acting antivirals (DAAs) have resulted in a sustained virological response (SVR) rate of 95–99% in treating HCV. Several studies implied that DAAs treatment may be related to increased risk of developing HCC in the first 6–12 months [21–23]. The molecular mechanisms of how DAAs increased the development of HCC have been investigated in several studies [27–32]. Giovannini et al., found the possible occurrence of off-target effects that modulated cell proliferation and/or migration and potentially justified previous findings showing some instances of particularly aggressive HCC recurrence as well as reduced incidence of HCC recurrence after DAAs treatment [44]. In another recent study, Ogawa et al., found that the annual incidence of the development of HCC within the first two years was higher for HCV patients treated with SOF than for those without, but did not reach statistical significance [45].

However, the involvement of SOF in tumor development of HCC remains unclear. This study obtained the following findings. First, SOF increased cell proliferation and metastatic characteristics in HCC cells with or without HCV virus. Second, NGS and TCGA data analysis revealed that several SOF-upregulated genes, including PHOSPHO2-KLHL23, TSNAX-DISC1, TRIM39 and RPP21, were also higher in tumor compared with non-tumor parts in patients with HCC. Third, knockdown of these genes ameliorated SOF-mediated cell proliferation and migration. The present results imply that SOF might upregulate these genes to facilitate tumor development of HCC.

SOF is the inhibitor of NS5B of HCV. The NS5B of HCV is a 68 kDa tail-anchored protein with 21 C-terminal amino acids alpha helical trans-membrane domain [46]. NS5B RNA-dependent RNA polymerase (RdRp) is the important component of the replicative complex and is critical for viral RNA replication and initiation [47, 48]. However, the clinical significance of SOF-upregulated genes in the carcinogenesis of HCC is largely unknown. This study found that several genes including PHOSPHO2, KLHL23, TSNAX-DISC1, TRIM39 and RPP21 were upregulated by SOF and the expression levels of these genes were higher in tumor than in non-tumor parts of patients with HCC according to the TCGA database. The present results indicated that several SOF-upregulated genes including PHOSPHO2-KLHL23, TSNAX-DISC1, TRIM39 and RPP21were associated with the development of HCC. Moreover, the findings also demonstrated that high level of PHOSPHO2 and RPP21 were correlated with poor overall survival of HCC, while high level of PHOSPHO2 was associated with disease-free survival. Silencing SOF-upregulated genes reduced significantly elevated cell proliferation and migration of HCC cells stimulated by SOF. Taken together, the SOF-upregulated genes may contribute to the development of HCC, the overall survival and disease-free survival of HCC. The present results may offer an explanation for the unexpected high recurrence rate of HCC in CHC patients undergoing DAA treatment [21–23].

This study also discovered that SOF increased cell proliferation and migration in hepatoma cells with or without HCV infection, while knockdown of PHOSPHO2-KLHL23, TSNAX-DISC1, TRIM39 and RPP21 reduced the increase in cell proliferation and migration by SOF in OR-6 (HCV-positive HCC cell line) and Huh 7.5.1 (HCV-negative HCC cell line) cells. Enhanced cell proliferation and migration were essential for tumor carcinogenesis. The present findings suggested that several SOF-upregulated genes were associated with tumor growth and metastatic characteristics, which might be associated with the occurrence of HCC and unfavorable prognosis regardless of whether the cells are infected with HCV or not. This is the first study indicating that SOF may be involved in the carcinogens of HCC via its upregulated genes. In Figure 3, the expression levels of phospho2-KLH23 and TSNAX-DISC1 were increased upon sofosbuvir treatment in a dose dependent manner and peaked at 1 μM of SOF. From the point of pharmacology, the effects of SOF may reach a plateau at a concentration of 1 μM and subsequently decreased at a concentration of 2.5 μM, which might be a normal pharmacological phenomenon.

Besides, cured OR-6 cells using either Interferon-α or DAA may actually influence the phenotype of OR-6 cells. Interferon-α or DAA treated OR-6 cells may not be a good control model for the study of SOF upregulated genes in OR-6 cells. Thus, the experiments on cured OR-6 cells were not performed.

Although OR-6 and Huh 7.5.1 cells have a cancer phenotype, this study found that several genes including PHOSPHO2, KLHL23, TSNAX-DISC1, TRIM39 and RPP21 were upregulated by SOF in OR-6 cells and the expression levels of these genes were higher in tumor than in non-tumor parts of patients with HCC according to the TCGA database. PHOSPHO2 and RPP21 were also found to be associated with overall survival of HCC patients in the TCGA database. Data from TCGA database, a human cancer database has confirmed our results and the prognostic roles of these were also revealed. And actually, recurrence of HCC was unexpectedly high among CHC patients with history of HCC treated with DAA [21, 22].

Several studies associated the candidate genes upregulated by SOF with carcinogenesis. A recent study found that overexpression of KLHL23 protein from read-through transcription of PHOSPHO2-KLHL23 increased cell proliferation in gastric cancer cells [49]. Another study found that lincRNA-NR_034037 influenced TSNAX-DISC1 formation that tightly regulate the development of endometrial carcinoma [50]. Trim39 was also found to be a biomarker for early diagnosis of ovarian cancer [51] and to regulate cell cycle progression and DNA damage responses [52]. The DNA damage signature including RPP21 can be employed to define a group of patients of prostate cancer with poor outcome and has the potential to be used as a prognostic marker in treatment [53]. Taken together, the SOF-upregulated genes have been found to be associated with the carcinogenesis in several cancers. Nevertheless, the comprehensive mechanisms of how these genes regulate the development of HCC require further studies to elucidate.

SOF increased the cell proliferation and migration on the HCC cell lines rather than the normal cells. The effects of hepatocarcinogenesis in chronic HCV patients with HCC treated with DAAs were controversial [18, 45, 54]. The association of the oncogenic implications of sofosbuvir and development of HCC require more studies to explain. In conclusion, results of this study suggested association of several SOF-upregulated genes with increased cell proliferation and migration, which may be associated with the development of HCC.

## MATERIALS AND METHODS

### Cell culture

Huh 7.5.1, OR-6 and AML-12 (alpha-mouse-liver-12) cells were used in this study. Huh 7.5.1 and OR-6 HCC cells are kindly provided by Dr. Francis Chisari and Dr. Nobuyuki Kato [55], respectively. OR-6 cells harbored full-length genotype 1b HCV RNA and co-expressed Renillaluciferase. AML12 mouse hepatocytes were purchased from Bioresource Collection and Research Center (60326™, BCRC, Taiwan). Huh 7.5.1 cells were grown in Dulbecco’s Modified Eagle’s Medium (DMEM) supplemented with 10% fetal bovine serum (FBS), while OR-6 cells were cultured with DMEM containing 10% FBS and 500 μg/ml of G418 (Promega, Madison, WI, USA). AML12 cells were cultured in DMEM and Ham’s F12 medium (1:1) supplemented with 10% FBS and antibiotics, including penicillin and streptomycin.

### Real-time PCR

After the identification of highly significant target genes, the primers for real-time PCR were designed with Primer Express 3.0 (Applied Biosystems, Foster City, CA, USA). Total cellular and viral RNA, isolated by RNeasy Mini columns (QIAGEN), were used to convert to cDNA with the High Capacity cDNA Reverse Transcription Kit (Applied Biosystems), followed by real-time PCR using the DyNAmo HS SYBR Green qPCR Kit (Finnzyme; Espoo, Finland). The primers for the genes are as follows: PHOSPHO2-KLHL23 forward 5′-GGAATAGTTGGGATGTGTTGCTT-3′ and reverse 5′-GAGTGTGGAATAGATGGTCTCACAGA-3′, TSNAX-DISC1 forward 5′-GGAAGATGCAGTTGAGAATGATGA-3′ and reverse 5′-TCTTGTTCCAGGTCTTCTAATCTTTG-3′, TRIM39 forward 5′-ACCACCACACCTTTTACCCC-3′ and reverse 5′-TATGAGAGCGGTCTGTGACAT-3′, RPP21 forward 5′-CTACACTGAGAGGACCATTGCG-3′ and reverse 5′-TGTTAGGCAGGTCTGTACGGT-3′, using GAPDH as normalization control.

### Cell viability assay

The effects of drugs on HCC cell viability were assayed as previously described [53]. Briefly, hepatoma cells were cultured in 96-well white plates for overnight and then treated with drugs for the time as indicated in the legends. The cellular ATP levels were measured with bioluminescent CellTiter Glo assay kit (Promega, Madison, WI, USA G7571) to monitor cell viability.

### Three-dimensional culture

Three-dimensional cell culture was performed as reported previously with minor modifications [56]. NanoCulture system was used for 3D cell culture (organogenix). First, 100 ul of medium was added to each well. The plate was then centrifuged at 2000 g for 5 min to remove microbubbles, followed by incubation for 15–30 min at incubator. Each well was seeded with 50 ul of medium containing 5 × 10^3^ cells (If needed, transfection was conducted at this time point.). The plate was then kept on the bench at room temperature for 10–15 min until cells adhered to the bottom film. At 24 h, 50 μl of medium (including drug) was added for 4–6 days until observation of sphere formation. Pictures of the spheres were taken under microscope and cell viability was measured with 3D Cell Titer Glo (Promega, Madison, WI, USA).

### Migration assay

Migration activity of HCC cells was evaluated with wound healing assays using IBIDI Culture-Inserts (35 mm with high culture-insert coating). First, HCC cells (1.5 × 10^5^ cells/140 μl medium) were plated in the culture insert for overnight to allow cells attach well. The cell debris were washed out with PBS to keep HCC cells in monolayers. The wound healing distance was used to access the migratory activity of HCC cells.

### Library preparation for transcriptome sequencing

A total amount of 1 μg RNA in each condition was used to generate libraries for transcriptome sequencing using TruSeq stranded mRNAlibrary prep Kit (cat# RS-122-2101, Illumina, San Diego, CA, USA) according to manufacturer’s recommendations. Briefly, mRNA was reverse converted to cDNA using SuperScript II Reverse Transcriptase and amplified with 2X PCR Master Mix. Adaptors were attached to DNA fragments and purified with AMPure XP system (Beckman Coulter, Beverly, MA, USA). The DNA High Sensitivity Chips were employed to determine quality of the library in Agilent Bioanalyzer 2100 system. The whole transcriptome were sequenced on an Illumina Next Seq 500 platform.

### Bioinformatics analysis

The bioinformatics analysis pipeline is followed from sequencing step. Low quality bases and sequencing adapters in raw data which generated from Illumina sequencer was eliminated. The DNA sequences were aligned to reference using Bowtie2 and the gene expression level was calculated by the Expectation Maximization (EM) algorithm for its statistical model. Differentially expressed genes (DEGs) are calculated by EBSeq, and the potential functions of those DEGs was accessed with GO and KEGG analysis.

### Gene knockdown

The scramble siRNA and siRNA oligonucleotides targeting candidate genes were purchased from Qiagen-Xeragon (Germantown, MD, USA) and mixed with Lipofectamine™ RNAiMAX Transfection Reagent (Invitrogen, Carlsbad, CA, USA) according to manual instruction. All siRNAs used for gene knockdown were SMART pools to minimize off-target effects and the silencing effects were determined by real-time PCR.

### Statistical analysis

The statistical results were expressed as mean ± SEM from at least three individual experiments. Results of cell proliferation assay and migration assay were analyzed using non-parametric two-tailed Student’s *t*-test. Gene expression and clinical datasets of HCC patients were obtained from TCGA database (https://cancergenome.nih.gov/) and analyzed with analysis of variance (ANOVA). Differential expression of candidate genes between the tumor tissues and adjacent non-tumor part was evaluated using the Wilcoxon signed-rank test. The receiver operating characteristic (ROC) curve were used to set the cutoff for high and low expression groups. Cumulative survival curves and adjusted hazard ratios for both overall and disease-free survival were evaluated with the Kaplan-Meier method and univariate and multivariate Cox proportional hazards models, respectively. A *p* value less than 0.05 was considered statistically significant.

### Availability of data and materials

The datasets used and analyzed during the current study are available from the corresponding author on reasonable request.

## Supplementary Materials

Supplementary Figures

Supplementary Table 1
