# Supplementary material for: Sofosbuvir induces gene expression for promoting cell proliferation and migration of hepatocellular carcinoma cells
Source: Aging (Albany NY). 2022 Jul 12;14(14):5710–26. doi: 10.18632/aging.204170 (PMC9365546; doi:10.18632/aging.204170)
Supplement: Supplementary Figures [file aging-14-204170-s001.pdf]

SUPPLEMENTARY FIGURES

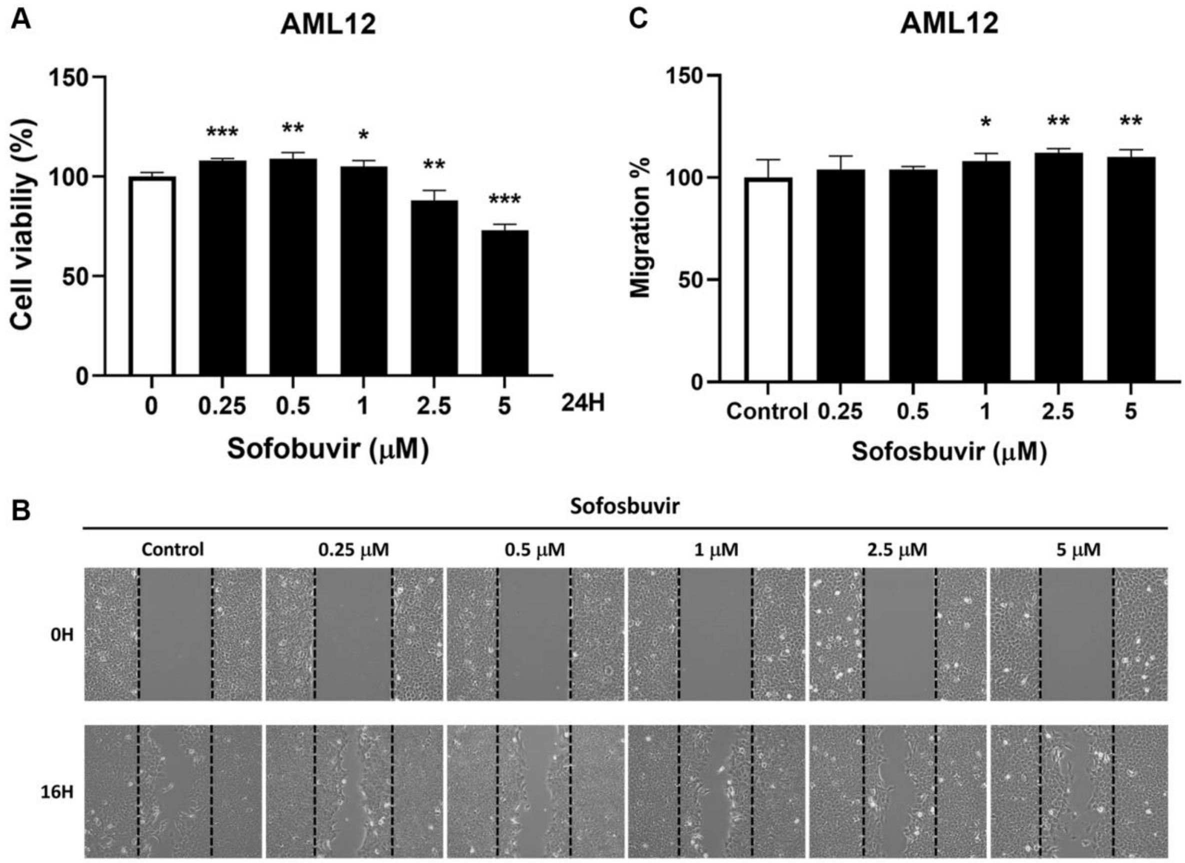

Supplementary Figure 1. The effects of sofosbuvir, on cell proliferation and migration assay in AML-12 cells. (**A**) Two-dimensional cell proliferation assay in AML-12 cells treated with different doses of SOF (0.25 to 5 μM) were performed. The experiments were performed from three independent experiments. ($^{*}p < 0.05$; $^{**}p < 0.01$; $^{***}p < 0.001$). (**B** and **C**) Migration assay of AML-12 cells treated with different doses of SOF (0.25 to 5 μM) were performed. The experiments were performed from three independent experiments. ($^{*}p < 0.05$; $^{**}p < 0.01$; $^{***}p < 0.001$).

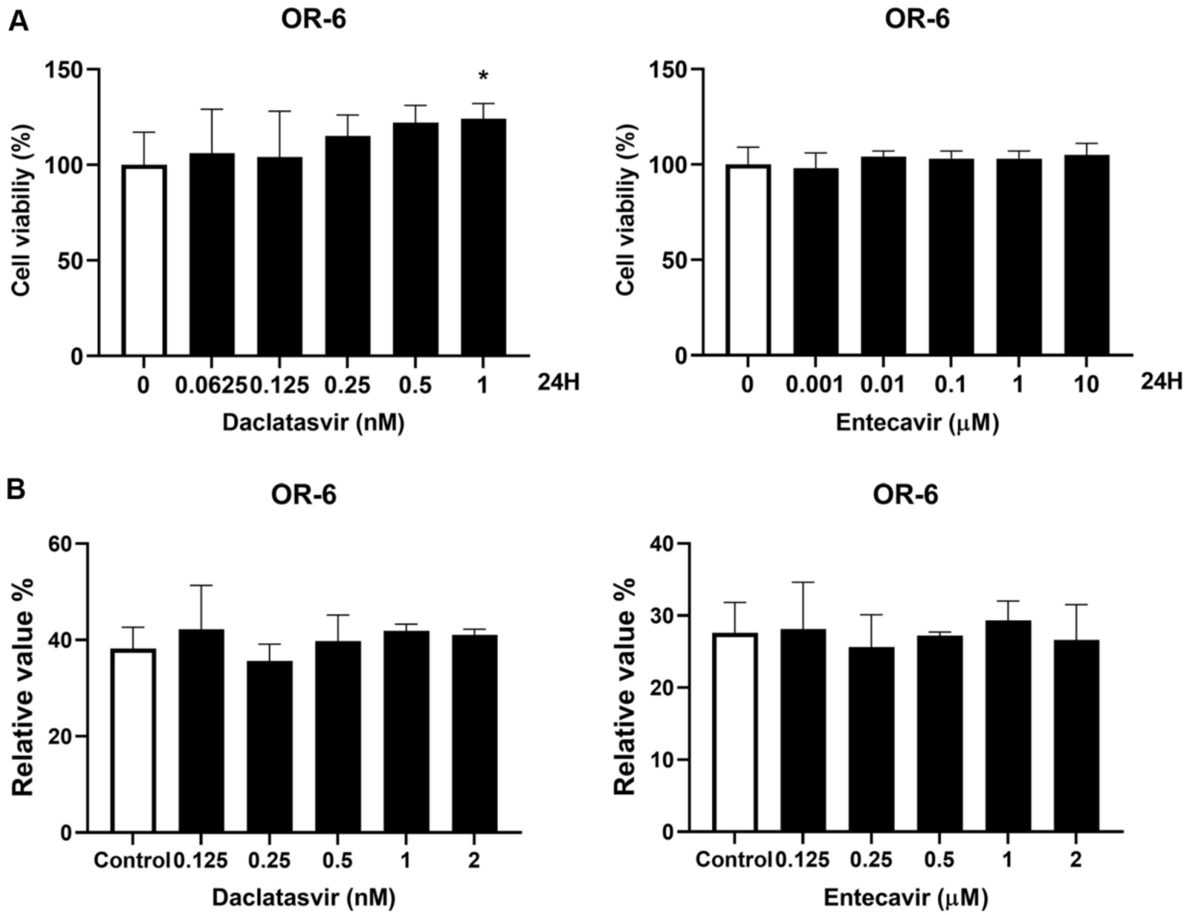

Supplementary Figure 2. The effects of daclatasvir and entecavir on cell proliferation and migration assay in OR-6 cells. (**A**) Two-dimensional cell proliferation assay in OR-6 cells treated with different doses of daclatasvir and entecavir were performed. The experiments were performed from three independent experiments. ($^{*}p < 0.05$; $^{**}p < 0.01$; $^{***}p < 0.001$). (**B**) Cell migration assay in OR-6 cells treated with different doses of daclatasvir and entecavir were performed. The experiments were performed from three independent experiments. ($^{*}p < 0.05$; $^{**}p < 0.01$; $^{***}p < 0.001$).

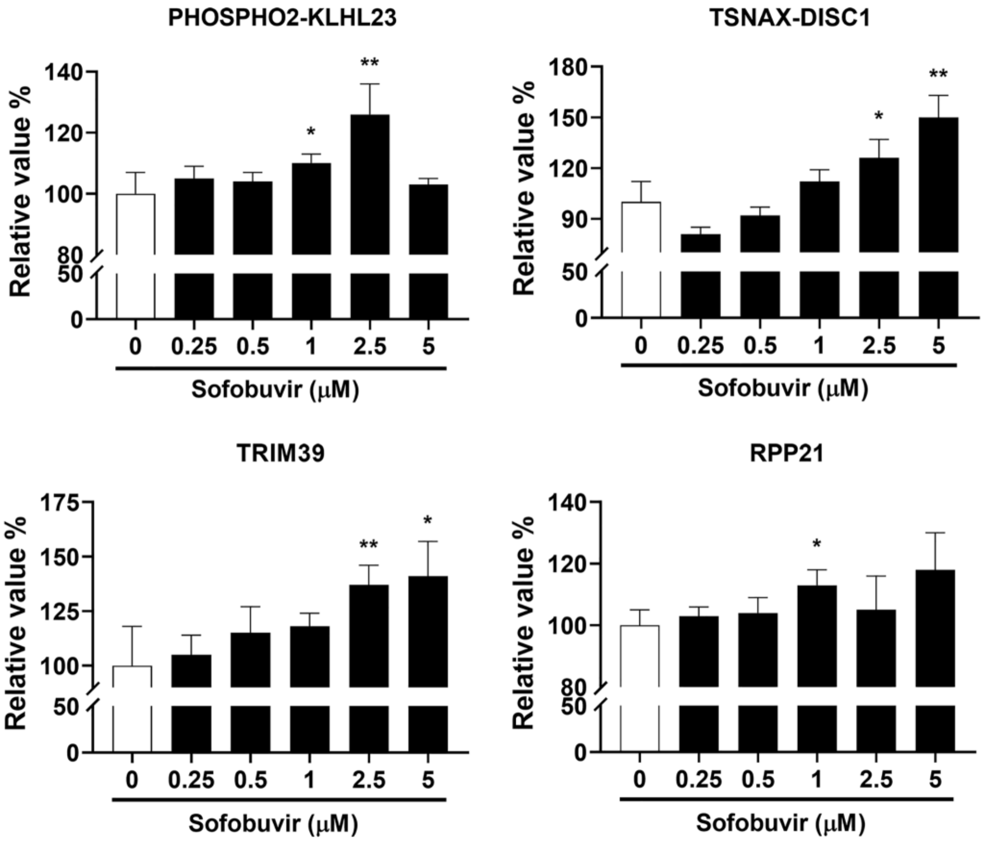

Supplementary Figure 3. Candiate genes up-regulated by SOF in Huh 7.5.1 cells. To avalidate the SOF-upregulated genes identified from NGS, the Huh 7.5.1 cells were treated with SOF (0.25 to 5 μM) for 24 hrs. The cells were harvested to determine gene expression with Q-PCR. Q-PCR validation of PHOSPHO2-KLHL23, TSNAX-DISC1, TRIM39, RPP21 in Huh 7.5.1 cells were performed using GAPDH as a normalized control. The experiments were performed from three independent experiments. ($^{*}p < 0.05$; $^{**}p < 0.01$; $^{***}p < 0.001$).
