# Supplementary material for: Sofosbuvir induces gene expression for promoting cell proliferation and migration of hepatocellular carcinoma cells
Source: Aging (Albany NY). 2022 Jul 12;14(14):5710–26. doi: 10.18632/aging.204170 (PMC9365546; doi:10.18632/aging.204170)
Supplement: Supplementary Table 1 [file aging-14-204170-s002.pdf]

SUPPLEMENTARY TABLE

Supplementary Table 1. Genes up-regulated by sofosbuvir in OR-6 cells.

Gene name	Ensembl Transcript ID	Gene type	Fold change	*P*-value
SMN2 (survival of motor neuron 2)	ENST00000380743.8	Protein coding	4181	<0.05
NPHP3-ACAD11	ENST00000632629.1	Protein coding	156	<0.05
UBE2F-SCLY	ENST00000449891.5	Nonsense mediated decay	148	<0.05
RALGAPAIP	NR_104269.1.1	Pseudogene	136	<0.05
WRB-SH3BGR	NM_001317744.1.1	Protein coding	106	<0.05
TRIM39-RPP21	ENST00000623385.3	Protein coding	91	<0.05
HOXA10-HOXA9	NR_037940.1.1	Misc RNA	82	<0.05
TMEM189-UBE2V1	ENST00000341698.2	Protein coding	66	<0.05
GOLGA6L17P	ENST00000611297.4	Pseudogene	62	<0.05
JMJD7-PLA2G4B	ENST00000382448.8	Protein coding	46	<0.05
CLEC18B	ENST00000339953.9	Protein coding	37	<0.05
SNORD141A	NR_132980.1.1	snoRNA	36	<0.05
SNORD141B	NR_132981.1.1	snoRNA	36	<0.05
PHOSPHO2-KLHK23	NM_001199290.1.1	Protein coding	17	<0.05
CTAGE9	ENST00000314099.9	Protein coding	13	<0.05
TGIF2-C20orf24	ENST00000558530.1	Protein coding	7	<0.05
ATP5J2-PTCD1	ENST00000413834.5	Protein coding	5	<0.05
NBL1	ENST00000375136.7	Protein coding	4	<0.05
RNASEK-C17orf49	ENST00000547302.3	Protein coding	3	<0.05
TSNAX-DISC1	ENSG00000270106	Protein coding	3	<0.05
C8orf44-SGK3	ENST00000519289.1	Protein coding	2	<0.05
POC1B-GALNT4	ENST00000548729.5	Protein coding	2	<0.05
ACOT1	ENST00000311148.8	Protein coding	2	<0.05
SERF2-C15ORF63	NR_037673.1.1	Misc RNA	2	<0.05
RPS10-NUDT3	ENST00000639725.1	Protein coding	2	<0.05
